# Preclinical robotic device for magnetic resonance imaging guided focussed ultrasound

**DOI:** 10.1002/rcs.2466

**Published:** 2022-10-01

**Authors:** Marinos Giannakou, Anastasia Antoniou, Christakis Damianou

**Affiliations:** ^1^ R&D Medsonic Ltd Limassol Cyprus; ^2^ Department of Electrical Engineering, Computer Engineering, and Informatics Cyprus University of Technology Limassol Cyprus

**Keywords:** focussed ultrasound, MRI guidance, preclinical research, robotic device

## Abstract

**Background:**

A robotic device featuring three motion axes was manufactured for preclinical research on focussed ultrasound (FUS). The device comprises a 2.75 MHz single element ultrasonic transducer and is guided by Magnetic Resonance Imaging (MRI).

**Methods:**

The compatibility of the device with the MRI was evaluated by estimating the influence on the signal‐to‐noise ratio (SNR). The efficacy of the transducer in generating ablative temperatures was evaluated in phantoms and excised porcine tissue.

**Results:**

System's activation in the MRI scanner reduced the SNR to an acceptable level without compromising the image quality. The transducer demonstrated efficient heating ability as proved by MR thermometry. Discrete and overlapping thermal lesions were inflicted in excised tissue.

**Conclusions:**

The FUS system was proven effective for FUS thermal applications in the MRI setting. It can thus be used for multiple preclinical applications of the emerging MRI‐guided FUS technology. The device can be scaled‐up for human use with minor modifications.

## 1 INTRODUCTION

Focussed ultrasound (FUS) therapy is a promising treatment method against various diseases.^1^ By focussing the ultrasonic beam, an increase in temperature is achieved due to the absorption of the ultrasonic energy by the tissue.^2^ Accordingly, local therapy is possible even for targets located deep in the body.^3^ The focal point is just a few mm in diameter depending on the transducer characteristics. This has the advantage of accessing targets with high precision and no damage on the surrounding tissue.^4^ So far, therapeutic ultrasound has been evaluated in multiple oncological^5^, ^6^, ^7^, ^8^ and neurological applications^9^, ^10^, ^11^ with very promising results. Due to its numerous benefits and wide range of potential applications, FUS play an important role in the future of medicine.

Magnetic Resonance Imaging (MRI) provides high resolution imaging of soft tissues. In addition, the imaging sequences used in MRI are temperature sensitive. Due to this property, it is possible to monitor the temperature evolution with the use of image processing.^12^ It is therefore the ideal diagnostic method for FUS guidance.^13^ MRI with fast imaging sequences can monitor temperature changes and estimate the delivered thermal dose during heating in almost real time.

Due to the small size of the focus, multiple overlapping lesions must be formed for ablating a large tissue volume. Thus, a robotic system is needed to accurately guide the transducer without intervention by the medical personnel, which would result in extremely long treatment sessions. Simultaneously, robotic operation offers the accuracy and precision required for such procedures, and thus, it is clearly safer. In addition, a robotic system allows treatment in a non‐sequential pattern, thus reducing the prefocal heating and treatment duration.^14^

Various companies are involved in the development of preclinical FUS systems. One of them is the FUS Instruments company^15^ owing two MRI compatible devices. The first one was specifically developed for 9.4 T MRI scanners, which have a small bore diameter.^16^ The second device is larger in size and is compatible with MRI scanners of 1.5–3 T.^17^ Image guided therapy is another company offering a wide range of products in the field of therapeutic ultrasound, including positioning systems.^18^ Another company known for its wide range of ultrasound research systems is Verasonics.^19^ This company offers a platform for FUS applications under diagnostic ultrasound guidance.^19^ Although ultrasound is cheaper and can be easily integrated to a robotic system, it has lower image quality and does not provide any temperature information.

The development of MRI‐compatible robotic systems is challenging due to the limitations related to the materials, motion actuators and encoders employed. A careful selection of materials and mechatronic components is required so that there is no significant interference with the scanner. In addition, the available space of the MRI scanner is very limited.^20^ Thereby, the device must be able to fit inside the MRI bore while allowing enough space for the patient.

An emerging application that is still in the preclinical phase and has already attracted the attention of the research community is the FUS‐mediated transient opening of the blood brain barrier (BBB).^21^ The permeability of the BBB to large molecules prevents most of the drugs from entering the brain tissue.^21^ Therefore, therapeutic drugs cannot normally reach the brain in the appropriate concentration to trigger the desired effect.

BBB opening could be beneficial in the treatment of numerous neurological diseases as it allows therapeutic agents to enter the brain parenchyma.^22^ With FUS it is possible to reversibly disrupt the BBB for several hours allowing sufficient drug delivery while maintaining its defensive mechanism unaffected.^22^ The benefits of FUS‐mediated BBB disruption were proven in numerous animal studies.^23^, ^24^ Most studies were conducted in rodents, which are usually easier in handling and require less expensive facilities.^25^, ^26^

Before a new device can be used in humans, it must be extensively evaluated ex‐vivo in phantoms and excised animal tissue, as well as in vivo in animals. The purpose of pre‐clinical trials is to extract data on the safety and the efficiency of the device and therapeutic protocol for the specific intended application. For this reason, there is a great interest from the research community for preclinical systems to accelerate the evaluation process of emerging applications in the field.

In prior studies, a lot of FUS robotic systems with varying functionalities and intended applications have been proposed.^27^, ^28^, ^29^, ^30^, ^31^, ^32^, ^33^, ^34^ Motion was established through different mechanisms including linear ball,^28^ brass racks and pinion,^29^ and jackscrew^34^ mechanisms. Both piezoelectric^28^, ^29^, ^31^, ^34^ and pneumatic^30^ motors were utilised for actuating motion. So far, our group manufactured numerous MRI‐guided FUS (MRgFUS) systems by 3D printing, which comprise piezoelectric motors and MR compatible optical encoders for precisely actuating and monitoring motion, respectively.^31^, ^32^, ^33^, ^34^

In the current study, we propose an in‐house developed robotic device with advanced ergonomics for preclinical studies on MRI‐guided FUS. The proposed device has compact dimensions, which make it capable for integration with all commercial scanners of cylindrical bore. Specifically, it can be sited on or fitted in the MRI table with the animal laying above an acoustic opening for ultrasonic coupling. The positioning mechanism actuates motion of the FUS transducer in the three cartesian axes. Movement in each axis is established by piezoelectric motors and controlled by a set of MR compatible optical encoders. Due to the non‐invasive nature of therapeutic ultrasound, recovery of the animals will be faster and postoperative pain will be minimised.

The main innovation of the system is its mechanical design that addresses the issue of water volume fluctuation during motion occurred in previously proposed systems,^27^, ^34^ thereby avoiding the use of vacuum mechanisms. Specifically, the transducer is actuated in a water container along with all the moving parts, whereas the motors and encoders are accommodated in a separate enclosure. The motion is transferred into the water container via shafts that are sealed using O‐rings to avoid water leakage to the motors' enclosure. The design of the various mechanical assemblies was proven challenging since they had to be compactly arranged in a single enclosure leaving sufficient space for the transducer to move. Special gear mechanisms and shaft guides were incorporated to achieve a smooth and reliable motion. The wide range of motion will enable adaptation of the system for human applications upon minor changes. Furthermore, in contrast to previously developed systems,^28^, ^29^, ^33^ the proposed one has all its electronic and mechanical components hosted in a single compact enclosure, thus offering improved safety and ergonomics. Another key benefit is the highly accurate motion achieved through the use of a set of optical encoders for each individual motion axis. The combination of all the aforementioned benefits makes the system unique.

## 2 MATERIALS AND METHODS

### 2.1 Focussed ultrasound (FUS) setup

The device comprises an in‐house manufactured piezoelectric transducer made out of non‐magnetic materials. A concave piezoelectric element with a frequency of 2.75 MHz, an active diameter of 50 mm, and a geometric focussing radius of 65 mm (Piezo Hannas Tech co. Ltd) was hosted in a plastic case and secured with epoxy (2‐part epoxy adhesive, Asonic). Note that the transducer specifications were selected to achieve a sharp beam focussing at sufficient depth in tissue following simulation of the FUS beam and heating effects of candidate transducers with varying characteristics (frequency, diameter, and radius of curvature).

The impedance of the transducer was matched to a high‐power amplifier (AG1016, AG Series Amplifier, T & C Power Conversion, Inc.) using a custom manufactured matching circuit. Its acoustic efficiency was experimentally determined by the radiation force balance method^35^ and found to be 30%. Based on the power capacity of the transducer the maximum depth that lesions can be created is 10 cm.

### 2.2 Positioning device

A robotic system with three degrees of freedom (DOF) was developed. The device manoeuvres the ultrasonic transducer in the *X*, *Y* and *Z* linear axes, with an available motion range of 80, 90, and 62 mm, respectively. Most of the device components were manufactured using a Fused Deposition Modelling (FDM) 3D printing machine (FDM 270, Stratasys). Some parts of the device that needed to have a highly accurate design and solid infill were manufactured using a polyjet 3D printing machine (Object30 pro, Stratasys). The FDM parts were made out of Acrylonitrile styrene acrylate (ASA) thermoplastic, whereas the polyjet parts were made out of VeroWhite resin material.

The robotic system utilises ultrasonic motors (USR60‐S3N, Shinsei Kogyo Corp.), whose motion is controlled by optical encoders (EM1‐2‐2500, US Digital Corporation) with a resolution of 2500 lines per 360°. The angular motion produced by the motors is converted into linear motion by jackscrew‐based mechanisms.

**FIGURE 1:**
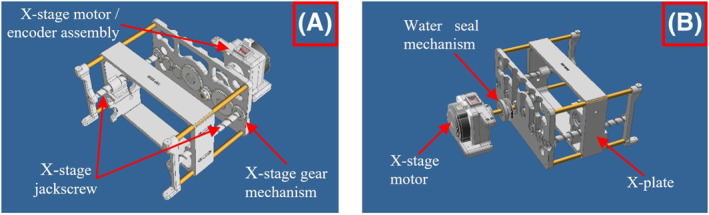
Computer‐aided design (CAD) drawing of the *X*‐stage mechanism: (A) Front view, (B) Rear view

The *X*‐stage is shown in Figure 1. The rotational motion of the *X*‐stage motor is transferred into the water container by a brass shaft, which rotates a gear mechanism. The gear mechanism was linked with the two jackscrews, which were in turn coupled with the *X*‐plate as shown in Figure 1. Rotation of the motor induces linear motion of the *X*‐plate along the respective jackscrews. Four guiding rods with a diameter of 8 mm were incorporated in the mechanism to ensure stable and smooth positioning in the *X*‐axis.

**FIGURE 2:**
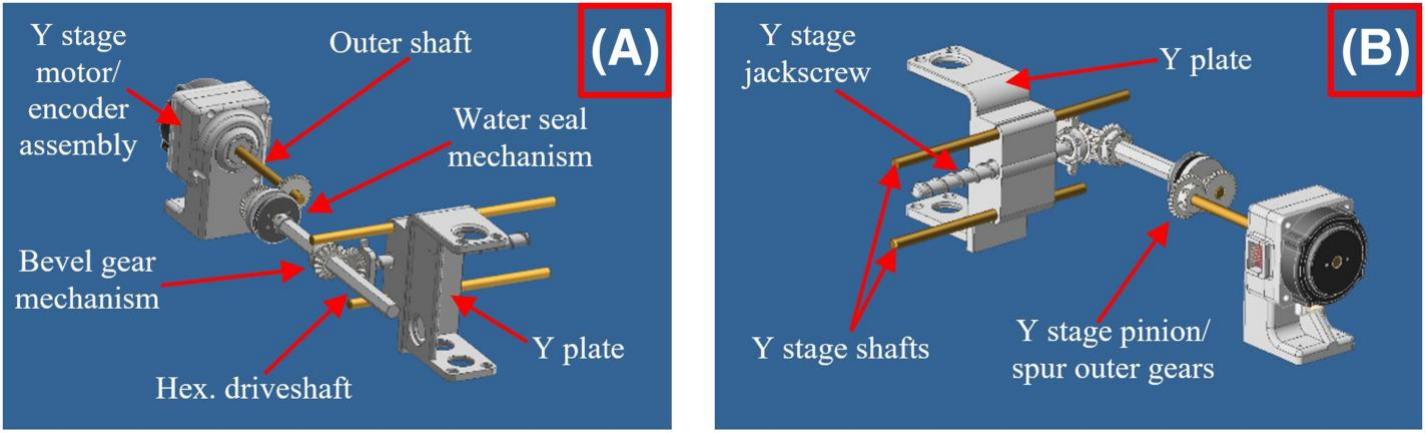
Computer‐aided design (CAD) drawing of the *Y*‐stage mechanism: (A) Front view, (B) Rear view

The *Y*‐stage shown in Figure 2 involves bevel gears coupled to a hexagonal driveshaft, thus transferring the motion at 90° (along the *Y* axis). During motion in the *X*‐axis, the bevel gears mechanism slides along the driveshaft following the *X*‐stage motion. During motion in *Y*‐axis, the gears rotate at a specific point in the *X*‐axis, thus transmitting the motion to the *Y*‐stage independently. Specifically, the *Y*‐stage motor as coupled to the hexagonal driveshaft rotates the bevel gears, which in turn rotate the *Y*‐stage jackscrew. Similar to the *X*‐stage, the *Y*‐plate is coupled to and moves along the respective jackscrew and two guiding rods.

**FIGURE 3:**
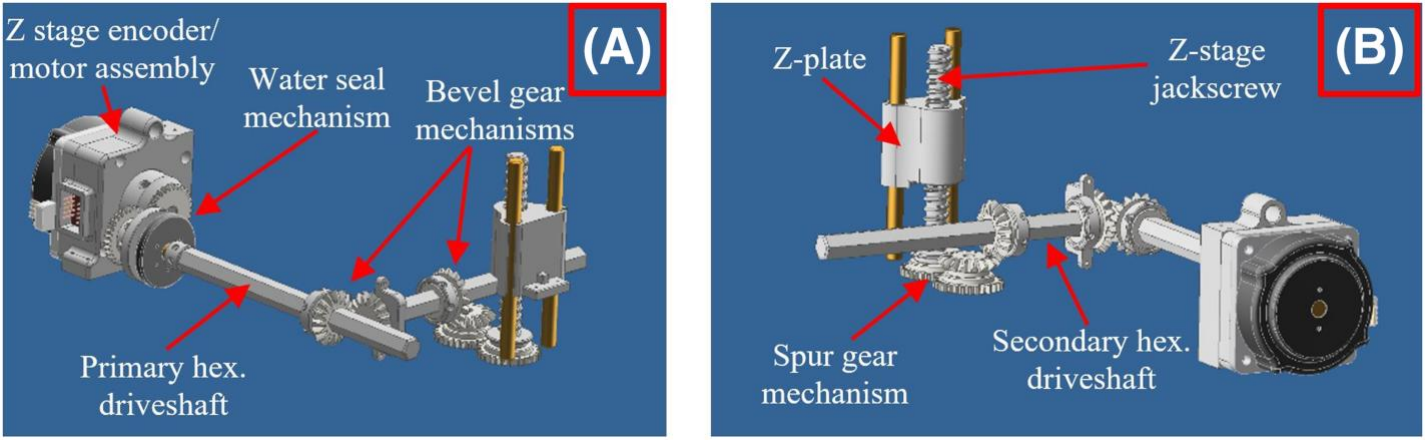
Computer‐aided design (CAD) drawing of the *Z*‐stage mechanism: (A) Front view, (B) Rear view

The *Z*‐stage has a more complex mechanism involving additional moving parts, as shown in Figure 3. This stage required the use of two hexagonal driveshafts so as to transfer the motion to the *Z*‐axis. Specifically, the *Z*‐stage motor was coupled to the primary hexagonal driveshaft rotating the first stage bevel gears. The first stage bevel gears were in turn coupled to the secondary hexagonal driveshaft, thus rotating the second stage bevel gears. The second stage bevel gears rotate a set of spur gears, which are located under the *Y*‐plate and are coupled to the *Z*‐stage jackscrew. Rotation of the jackscrew causes motion of the *Z*‐plate in the vertical direction along two guiding rods. With this configuration, the *Z*‐stage is able to move independently from the *X*‐stage and *Y*‐stage. The FUS transducer is attached to the respective coupling of the *Z*‐plate.

**FIGURE 4:**
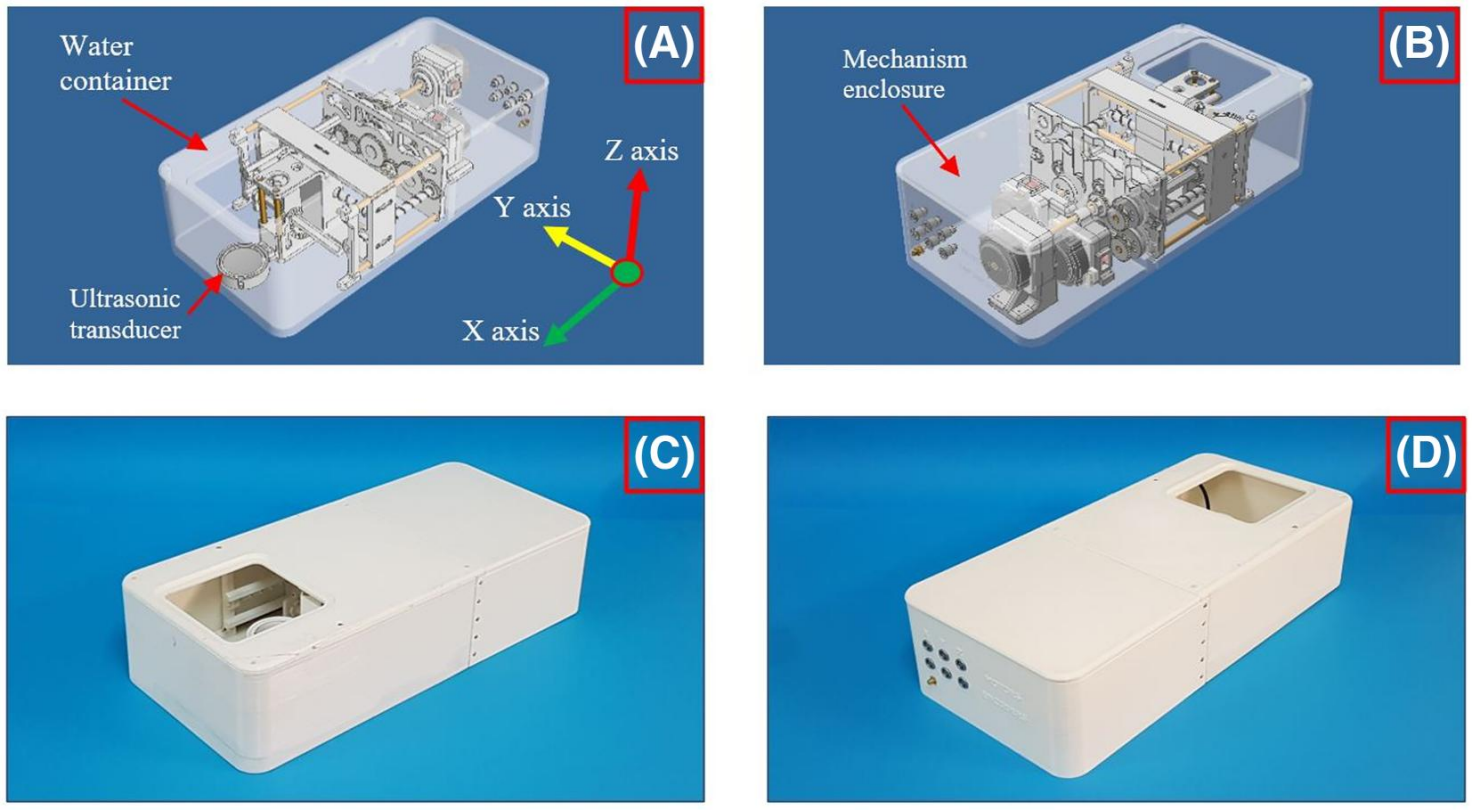
Computer‐aided design (CAD) drawing of the assembled robotic device with transparent covers: (A) Front view, (B) Rear view, and photos of the manufactured device: (C) Front view, (D) Rear view

Figure 4A,B show Computer‐aided design (CAD) drawings of the assembled robotic system. The moving parts were placed inside the water container, whereas the motors were placed in a separate mechanism enclosure located behind the water container. A simple and reliable mechanism with an O‐ring was used in each axis to seal the water container since ultrasonic motors cannot operate in water. The main advantage of placing the moving parts inside the water container is that water level fluctuation during positioning is prevented. Figure 4C,D show photos of the manufactured device.

The device is compact with a length of 50 cm, a width of 23 cm, and a height of 13 cm. Therefore, it can be placed in the table of all conventional scanners up to 7T. The patient lies above the device with the ultrasound reaching the target from bottom to top via the acoustic opening. Since a part of the device protrudes above the table, a mattress will be added forming a comfortable flat bed for the animal or patient in potential future clinical applications. Note that the mattress is placed around the device and not between the device and subject under test.

The hardware is interfaced with a controlling software that allows for remote control of the FUS system and robotic motion. Multiple sonications in grid and irregular patterns can be executed following path planning. The software also implements algorithms for treatment planning on pre‐operative MR images and monitoring of ultrasonic exposures through MR thermometry.^36^

### 2.3 Evaluation of the system

#### 2.3.1 Accuracy and repeatability of robotic motion

The robotic device was initially assessed in terms of the accuracy of positioning. Evaluation was done in the benchtop setting using a high precision digital calliper. The method was based on comparing specific steps (1, 5, and 10 mm) commanded through the controlling software with the actual displacements of the motion stage as estimated by the calliper. A detailed description of this calliper‐based technique can be found in previous work of our group.^37^

#### 2.3.2 Phantom preparation

An agar‐based phantom was prepared with 6% weight per volume (w/v) agar (Merck KGaA, EMD Millipore Corporation) as described in a previous study.^38^ The selection of agar was based on the fact that agar‐based phantoms can be easily prepared at low cost and have tissue‐like MRI signal.^39^ Additionally, this phantom has similar acoustical properties as human tissue.^39^, ^40^ The phantom was specially designed to securely fit the acoustic opening of the device so that vibrations during ultrasonic heating are minimised.

The phantom was used for assessing the MRI compatibility of the robotic device and heating ability of the FUS transducer using MR thermometry. Notably, image homogeneity in the MRI was achieved by continuous agitation of the agar mixture during preparation.^39^

#### 2.3.3 MRI compatibility

The robotic device was placed on the bed of a 1.5 T MRI scanner (GE Signa HD16, General Electric, Fairfield). The phantom was fitted in the acoustic opening. A body coil (Signa 1.5T 12 Channel, GE Healthcare Coils) was placed above the phantom using a custom‐made positioner made out of Polylactic acid (PLA) thermoplastic. The MRI compatibility of the system components was evaluated by estimating the influence on the Signal to noise ratio (SNR).

Images of the agar phantom were acquired under different activations of the positioning device using a Spoiled Gradient Echo (SPGR) sequence with the following parameters: repetition time (TR) = 22 ms, echo time (TE) = 10.5 ms, field of view (FOV) = 28 × 28 cm^2^, matrix = 192 × 160, flip angle = 30° and number of excitations (NEX) = 2. Image acquisition was performed with the cables disconnected (reference), cables connected, and DC ON (i.e., electronic system activated). Accordingly, the compatibility of the transducer with the scanner was evaluated by comparing SPGR images acquired with the amplifier activated (zero power applied) and electric power applied using the following parameters: TR = 22 ms, TE = 8.4 ms, FOV = 28 × 28 cm^2^, matrix = 192 × 160, flip angle = 30° and NEX = 2. In each case, the SNR was calculated as follows:

(1) $$SNR=\frac{{SI}_{phantom}}{\sigma_{noise}}$$

where the nominator represents the mean signal intensity (SI) of a region of interest (ROI) in the agar phantom and the denominator represents the standard deviation from the background ROI.

#### 2.3.4 MRI evaluation of thermal heating

The developed phantom was also used for evaluating the heating abilities of the FUS transducer. The transducer was fitted in a special plastic holder facing towards the bottom surface of the phantom. This setup was fitted in a water‐filled tank to achieve proper ultrasonic transmission. The tank was sited on the MRI scanner and phantom sonications were performed. MR thermometry maps were extracted by comparing 2D SPGR images acquired using the following parameters: TR = 22 ms, TE = 8.4 ms, FOV = 28 × 28 cm^2^, matrix = 192 × 160, flip angle = 30° and NEX = 2, according to the proton resonance frequency shift (PRFS)‐based technique previously described in detail by Menikou et al.^41^, ^42^ This method takes advantage of the change in the resonance frequency of water protons upon heating. The phase difference between a baseline image $\varphi \left({Τ}_{0}\right)$ and an image acquired at a specific time during heating φ(Τ) is proportional to the corresponding PRFS and it can be easily converted to temperature change as follows:^43^

(2) $$\Delta Τ=\frac{\varphi \left(Τ\right)-\varphi \left({Τ}_{0}\right)}{\gamma \alpha {Β}_{0}ΤΕ}$$

where γ is the gyromagnetic ratio, α is the PRF change coefficient, ${Β}_{0}$ is the magnetic field strength, and ΤΕ is the echo time. The range of temperatures (from a minimum to a maximum value) as calculated by MR thermometry were colour‐coded by adjusting a colour map from blue to red.

#### 2.3.5 Lesion creation in excised tissue

The effectiveness of the transducer in terms of thermal ablation was then evaluated by sonicating freshly excised porcine tissue. The piece of freshly excised porcine tissue was fitted to the acoustic opening above the FUS transducer, which was moved in grid patterns with a 60 s time delay and varying spatial step. Each spot was sonicated using electric power of 150 or 200 W for a duration of 10–30 s at a focal depth of 25 mm.

## 3 RESULTS

### 3.1 Accuracy and repeatability of robotic motion

Motion steps of 1, 5, and 10 mm were tested. The maximum mean positioning error (*n* = 10) occurred at the 1 mm step and was 0.044 ± 0.019 mm, 0.051 ± 0.023 mm, and 0.072 ± 0.034 mm for motion in the *X*, *Y*, and *Z* axes, respectively. These results demonstrate high accuracy and repeatability of robotic motion in all incorporated axes, with a maximum positioning error of about 0.1 mm.

### 3.2 MR compatibility

**FIGURE 5:**
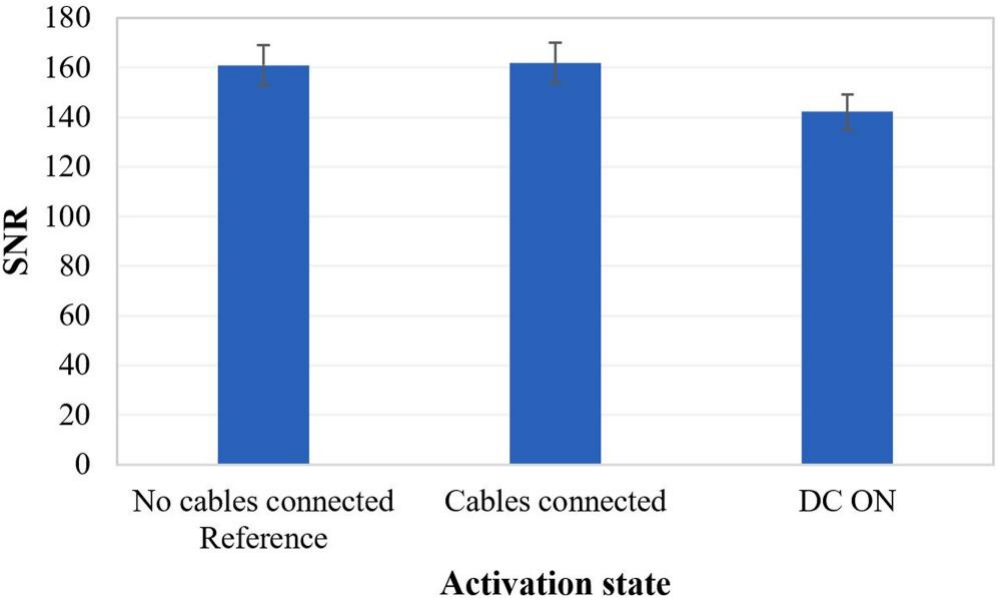
Signal‐to‐noise ratio (SNR) measurements from spoiled gradient echo (SPGR) phantom images acquired under different activation states of the positioning mechanism (MR parameters used: TR = 22 ms, TE = 10.5 ms, FOV = 28 × 28 cm^2^, matrix = 192 × 160, flip angle = 30° and NEX = 2)

The effect of activating different system components on the SNR was calculated. Initially, the phantom was imaged with all its electronics deactivated. At this condition, the highest SNR value of 161 was recorded providing the reference value for comparison with the different activations tested, as shown in the graph of Figure 5. Connection of the cables to the electronic driving system did not affect the image quality since the estimated SNR value was almost equal to the reference value. Activation of the DC supply dropped the SNR to approximately 142.

**FIGURE 6:**
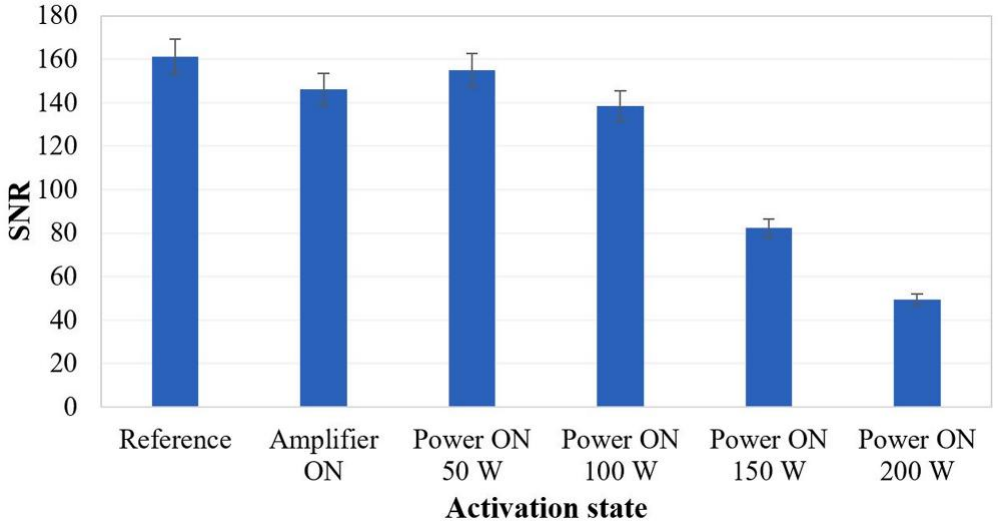
Signal‐to‐noise ratio (SNR) measurements from spoiled gradient echo (SPGR) phantom images acquired under different activation states of the focussed ultrasound (FUS) transducer (MR parameters used: TR = 22 ms, TE = 8.4 ms, FOV = 28 × 28 cm^2^, matrix = 192 × 160, flip angle = 30° and NEX = 2)

Next, the impact the transducer's activation has on the image quality for different electric power levels was investigated, as shown in Figure 6. Initially, the amplifier was activated (zero output power) resulting in an SNR value of 146, which is similar to that obtained when the positioning mechanism was activated (Figure 5). For electric power values of 50–200 W the estimated SNR values were in the range of 155–50 (respectively). The amplifier's activation seemed to introduce noise in almost linear fashion as the power increases (50–200 W).

### 3.3 MRI evaluation of thermal heating

**FIGURE 7:**
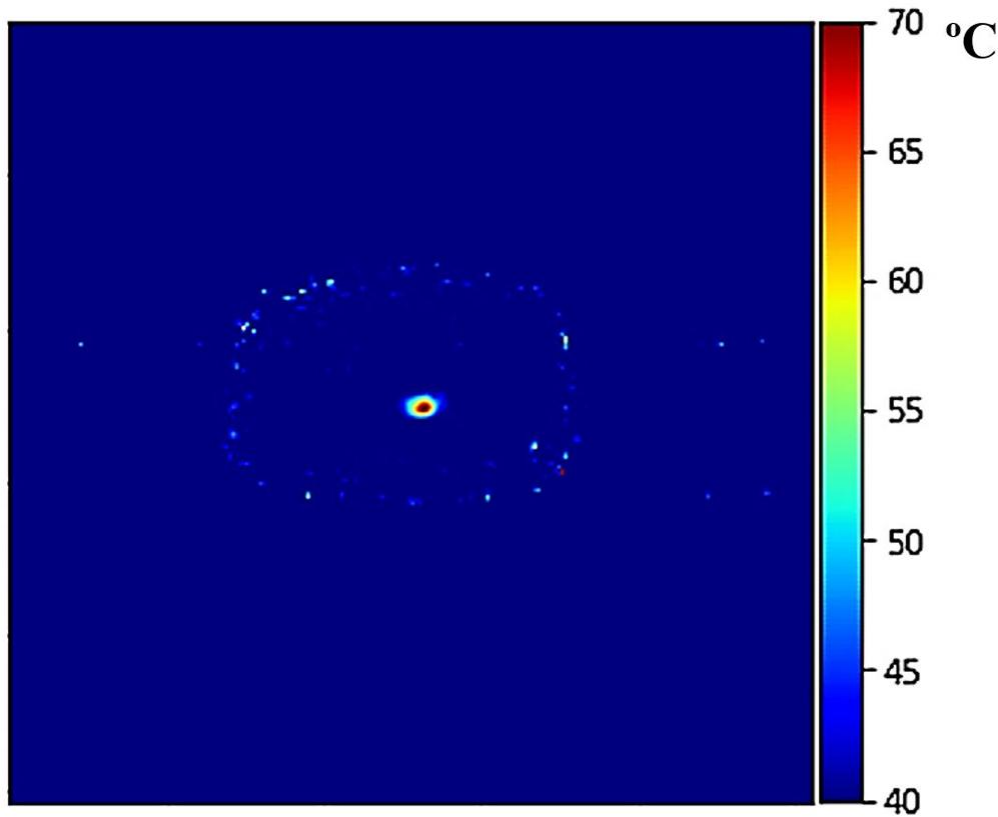
Coronal MR thermal map obtained in the focal plane at 50 s of sonication with electric power of 150 W using the spoiled gradient echo (SPGR) sequence (transducer specifications: frequency = 2.75 MHz, radius of curvature = 65 mm, diameter = 50 mm)

Thermal maps were generated using SPGR images of the phantom acquired every 7 s. Figure 7 shows a thermal map constructed at 50 s of sonication at electrical power of 150 W in a plane perpendicular to the ultrasonic transmission (coronal), indicating a peak temperature of about 70°C at the focal spot (baseline temperature of 37°C).

### 3.4 Lesion creation in excised tissue

**FIGURE 8:**
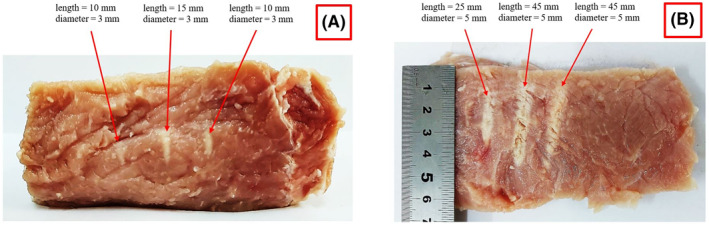
Photo of vertically dissected porcine meat showing lesions that were formed (on a plane parallel to the beam) in a 3 × 1 grid with a 20 mm step using electric power of (A) 150 W for 15 s, (B) 200 W for 20 s, at a focal depth of 25 mm (transducer specifications: frequency = 2.75 MHz, radius of curvature = 65 mm, diameter = 50 mm). Lesion's dimensions are indicated

Discrete lesions were initially produced on freshly excised porcine tissue. Figure 8A shows the lesions induced using electric power of 150 W for 15 s at the focal depth in tissue of 25 mm. Sequential sonications were performed in a 3 × 1 grid using a spatial step of 20 mm with a time delay of 120 s. Tissue was cut vertically (parallel to the ultrasonic beam) through the centre of lesions. The lesion diameter was approximately 3 mm and their length ranged from 10 to 15 mm. Note that the intervening tissue between the top surface of the meat and the focal depth does not seem to have been affected.

The lesions shown in Figure 8B were created using higher electric power of 200 W applied for a longer duration of 20 s while keeping the spatial and temporal step constant at the same focal depth (25 mm). In this case, the inflicted lesions were larger due to the increased power and were shifted towards the top surface of the meat. They had a larger diameter (approximately 5 mm) and a length in the range of 25–45 mm. The variation in lesion length is assumed to be the result of uneven tissue surface or other inhomogeneities and trapped air bubbles.

**FIGURE 9:**
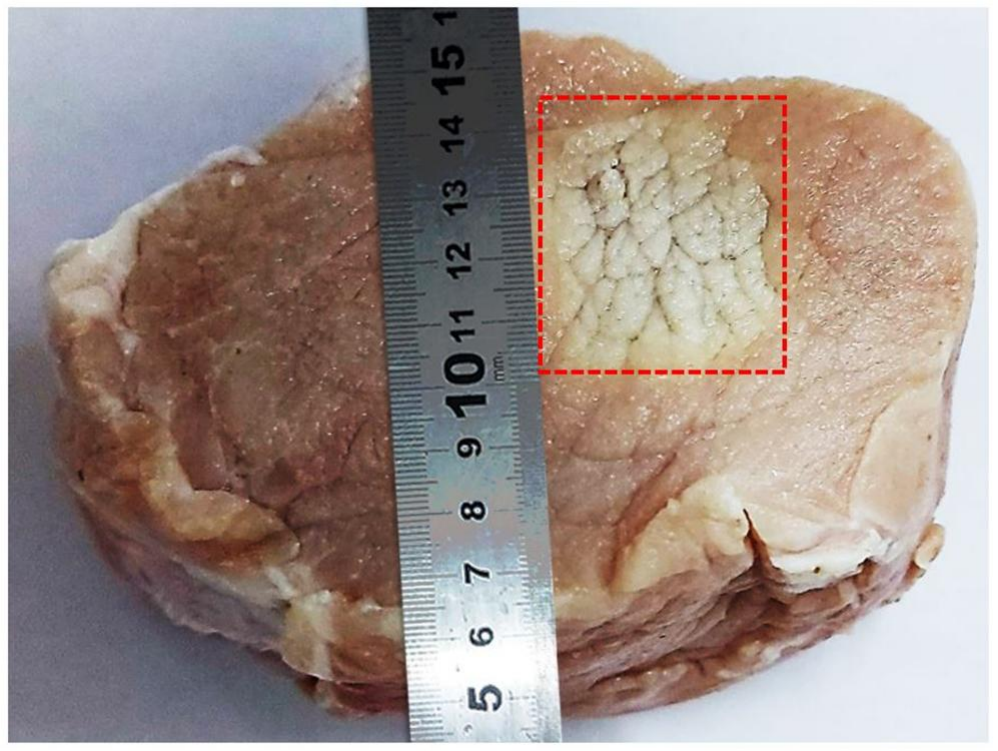
Photo of the top surface of excised meat after sonication in a 4 × 4 grid with a 4 mm step (60 s time delay) at a 25 mm focal depth using electric power of 200 W for 30 s at each spot (transducer specifications: frequency = 2.75 MHz, radius of curvature = 65 mm, diameter = 50 mm)

Sonications of similar electric power (200 W) applied for 30 s in a 4 × 4 grid with a smaller spatial step of 4 mm (60 s time delay) resulted in 16 overlapping lesions in tissue. Figure 9 shows the top surface of the meat where the ablated tissue covers an area of approximately 40 × 40 mm^2^.

## 4 DISCUSSION

A 3‐DOF robotic device was developed to facilitate preclinical research on MRgFUS. The FUS transducer and all the mechanical assemblies are actuated in a water container, whereas the motion actuators and controllers are hosted in a separate enclosure located at the rear of the device. This allows easy access to the mechanical and electronic components of the system. Piezoelectric motors are used for motion actuation. Note that this type of motors was widely used in the development of MRI compatible FUS devices.^27^, ^34^, ^44^, ^45^, ^46^, ^47^, ^48^ The angular motion of the motors is transmitted inside the water container via sealed shafts.

The robotic mechanism was specially designed to prevent water volume changes in the container during motion. By placing the motion stages inside the water container, fluctuation of the water volume is prevented since the mechanical parts are always occupying the same space. This approach eliminates the need for a bellow, which was used in previous studies to seal the coupling between the water container and the mechanism enclosure.^27^, ^34^ The bellow displaces the water especially during forward and reverse motion (*X*‐axis motion); hence the water container should include a vacuum system, thereby complicating the system's design and use.

The device is intended to be used in the MRI environment; hence magnetic materials were not incorporated. To ensure safe operation of the device inside the strong magnetic field of the scanner, several experiments were carried out. The SNR was the main metric for evaluating the effect of the system's activation on image quality. The acquired SNR values suggest that the quality of the SPGR images was not affected significantly by the presence of the device in the imaging field of the scanner, and thus the incorporated materials were considered appropriate. Activation of the various electronics (i.e., motors and encoders) did not seem to impact the SNR considerably as the SNR measurements were close to the reference value of 161. Noticeable SNR reduction occurred when electric power was applied. The SNR reduced gradually from about 155 to 50 with increasing electric power from 50 to 200 W. Note that a 3‐fold SNR reduction occurred at the highest acoustic power of 200 W. This is most probably attributed to the intense phantom vibrations occurring during intense heating. However, the SNR remained sufficiently high for the acquisition of thermal maps using MR thermometry algorithms. Note that the effect of power on image quality could be reduced substantially in higher field MRI scanners (3 and 7T), thus enabling the acquisition of high resolution images even at high power sonications.

The compact dimensions of the robotic device allow its placement in any commercial MRI scanner. The only requirement is to fit in the bore of the scanner. Due to its low weight (5.5 kg), it can be easily transported from the laboratory to the MRI setting. In addition, it can be easily prepared for use in a matter of few minutes. After use, it can be stored away as it is not integrated into the MRI bed permanently. Furthermore, the system is easy to be operated by the users.

Regarding future applications in humans, the 13 cm height of the device allows placement of humans for 3 and 7T scanners since the bore diameter is wide enough. For a 1.5 T scanner, the size of the robot would have to be reduced in order to accommodate humans with this robotic system. Basically, this design can be potentially fitted in all the scanners up to 7 T. For mice‐dedicated scanners (9.4 T), the space available when the coil is inserted is only 6–7 cm, and thus this device cannot be hosted in such scanners. It is also clarified that the device cannot be used in combination with the head coil, since the subject under preclinical testing should be placed above the acoustic window. Therefore, only surface type coils can be used with this device.

The FUS system was then tested for its effectiveness in producing sufficient heating using MR thermometry maps. Initially, low power sonication was performed to detect the focus location where the peak temperature occurs. Thermal maps were then acquired at the focal plane during intense heating, demonstrating the ability of the transducer to induce lethal temperature in the agar phantom without any recorded self‐heating effects.

After confirming efficient performance of the FUS transducer, the device was evaluated for its ability to produce thermal lesions in grid patterns through ex‐vivo experiments. Multiple sonications were performed in freshly excised porcine tissue using the automatic grid operation of the software. Discrete and overlapping lesions were successfully created in tissue. Unlike agar‐based phantoms, lesions in tissue are permanent. The production of lesions suggests that the temperature reached lethal levels, which is the main goal in oncological applications.

Discrete lesions were consistently created at the focal depth of 25 mm having similar diameter and length. This is a good indication of the thermal dose consistency and targeting accuracy of the device. Furthermore, the almost equal spacing arrangement of the formed lesions indicates high accuracy and repeatability of motion. Accurate motion is largely due to the high tolerances on the guides and stable driving mechanisms, as well as to the incorporation of a set of optical encoders on each axis that verify each other's operation.

It is interesting to note that the lesion size was proportional to the applied acoustic energy. Specifically, it was observed that an increase in the applied acoustic energy from 2250 to 4000 J (while keeping the other sonication parameters constant) resulted in discrete lesions of bigger dimensions, with a more than 2‐fold increase in lesion length. Further increase of the acoustic energy to 6000 J resulted in overlapping lesions and the creation of a single homogeneous ablation area. These experiments also proved that the system offers proper coupling with the target, as well as reliable isolation between water container and electronic parts (motors and encoders) since no water leakage was observed.

It is also worth noting that a small variability in the size of adjacent lesions was observed. This is most probably attributed to tissue inhomogeneities and the presence of fat layers that cause scattering and phase aberrations, thus affecting the ultrasonic propagation and penetration depth. It is also possible that air bubbles are trapped in the tissue causing intense acoustic reflection also affecting the formation of uniform lesions.

The intended applications of the system include testing and optimising therapeutic protocols, as well as assessing the performance of FUS software and treatment algorithms in the preclinical setting; in tissue‐mimicking phantom, excised tissue, and experimental animals. The available motion range is sufficient for the FUS beam to reach both shallow and deep tissue in animals of small to large size. Regarding BBB studies, a special holder could be fixed to the acoustic opening to accommodate rodents above the FUS transducer. Note that three DOF are more than enough for targeting the mouse brain, given its very small volume. However, the transducer should be replaced with one of proper characteristics for the specific application of BBB opening in mice. Typically, an operating frequency close to 1 MHz is suitable for minimising energy losses due to the skull. It should be also clarified that BBB opening is based on the mechanical (non‐thermal) effects of pulsed FUS.

The proposed device constitutes an evolution of previously proposed robotic systems.^27^, ^34^ Drakos et al.^34^ developed an MRgFUS robotic system for similar use. However, this device comprises a bellow for water sealing, which unavoidably induces water level fluctuations during robotic motion. As previously explained, the device proposed herein has a novel design that address this issue offering advanced ergonomics. In addition, it offers smoother motion that is mainly attributed to the use of gear mechanisms. Potential disadvantages of the system compared to the one proposed by Drakos et al.^34^ are its greater height and the lack of an angular motion stage. Note that an angular stage could be easily added in the positioning mechanism, but at the cost of increased complexity. Although angular motion of the transducer offers access to more challenging locations (e.g., behind the ribs), it complicates the system and might not be necessary for preclinical use. The next evaluation step is to test the device in animals, such as rabbits.

## 5 CONCLUSIONS

In summary, the current study proposes a robotic device with advanced ergonomics intended for preclinical research on the MRgFUS technology. The motion accuracy and MRI compatibility of the system in terms of proper imaging and thermal maps acquisition were demonstrated. The FUS system was proven safe and effective for thermal applications through MR thermometry experiments and visual assessment of lesion formation in excised porcine tissue. Overall, the results showed accuracy and consistency in the performance of the developed system throughout the sonication process. Further ex‐vivo and in vivo experiments in animals are needed to identify any malfunctions of the system and optimise the therapeutic protocol for applications in animals with cancer.

## AUTHOR CONTRIBUTIONS

Marinos Giannakou contributed to the development of the robotic device and draughting of the manuscript. Anastasia Antoniou contributed to the draughting of manuscript and implementation of the scientific methods. Christakis Damianou supervised the overall study, as well as the draughting of the manuscript.

## CONFLICT OF INTEREST

Marinos Giannakou declares no conflict of interest. Anastasia Antoniou declares no conflict of interest. Christakis Damianou declares no conflict of interest.

## Data Availability

The data that support the findings of this study are available from the corresponding author upon reasonable request.
